# The orthologue of the "acatalytic" mammalian ART4 in chicken is an arginine-specific mono-ADP-ribosyltransferase

**DOI:** 10.1186/1471-2199-9-86

**Published:** 2008-10-14

**Authors:** Andreas Grahnert, Steffi Richter, Fritzi Siegert, Angela Berndt, Sunna Hauschildt

**Affiliations:** 1 University of Leipzig, Institute of Biology II, Department of Immunobiology, Talstrasse 33, 04103 Leipzig, Germany; 2 Institute of Molecular Pathogenesis, Friedrich-Loeffler-Institute, Naumburger Str. 96a, 07743 Jena, Germany

## Abstract

**Background:**

Human ART4, carrier of the GPI-(glycosyl-phosphatidylinositol) anchored Dombrock blood group antigens, is an apparently inactive member of the mammalian mono-ADP-ribosyltransferase (ART) family named after the enzymatic transfer of a single ADP-ribose moiety from NAD^+ ^to arginine residues of extracellular target proteins. All known mammalian ART4 orthologues are predicted to lack ART activity because of one or more changes in essential active site residues that make up the R-S-EXE motif. So far, no other function has been detected.

**Results:**

Here we report the identification and characterisation of ART4 in chicken, which to our knowledge is the first true non-mammalian orthologue of a mammalian ART family member. The chicken *ART4 *gene has the same physical structure as its mammalian counterparts (three coding exons separated by two introns in phase 0 and phase 1, respectively) and maps to a region of conserved linkage synteny on chromosome 1. Its mRNA encodes a 289 amino acid protein with predicted N-terminal signal peptide and C-terminal GPI-anchor sequences and 47% sequence identity to human ART4. However, in striking contrast to its mammalian orthologues, the chicken protein contains an intact R-S-EXE motif. Upon ectopic expression in C-33A cells, recombinant chicken ART4 localized at the cell surface as a GPI-anchored, highly glycosylated protein, which displayed arginine-specific ART activity (apparent K_m _of the recombinant protein for etheno-NAD^+ ^1.0 ± 0.18 μM).

**Conclusion:**

The avian orthologue of the "acatalytic" mammalian ART4 is a mono-ADP-ribosyltransferase with enzymatic activity comparable to that of other, catalytically active and GPI-anchored members of the mammalian ART family.

## Background

ART4 is structurally related to vertebrate ecto-ADP-ribosyltransferases (ARTs), which covalently modify extracellular substrates by transferring a single ADP-ribose residue from NAD^+ ^to a specific amino acid in the target protein [1,2].

The mammalian ART family which comprises five members (ART1 – ART5) has been extensively studied in mice and humans [3]. In contrast to humans, which lack *ART2 *expression due to the presence of a non-functional *ART2 *gene [4], two ART2 proteins are expressed in mice as a result of gene duplication [5]. Among the five known ARTs, only ART1, ART2 and ART5 exhibit arginine-specific enzyme activity. ART3 and ART4 appear to have lost their catalytic activity, most likely due to the non-conservative substitution of residues in the R-S-EXE motif, which is typically present in the active centre of arginine-specific ARTs [6].

So far, no evidence for a potential other function has emerged for either of the two "acatalytic" members of the mammalian ART family, except that human ART4 has previously been demonstrated to be identical with the polymorphic Dombrock blood group antigen expressed on erythrocytes as GPI (glycosyl-phosphatidylinositol)-anchored glycoprotein [7,8]. In addition, we and others have shown that expression of the human *ART4 *gene can be induced by lipopolysaccharide, lipoteichoic acid and peptidoglycan in monocytes and alveolar epithelial cells [9-11].

Given the fact that the *ART2 *gene locus shows considerable genetic variation even among mammalian species (one active gene in rat, two active genes in mice versus one pseudogene in humans [4,5,12]), we sought to employ bioinformatic means to search for other *ART4 *orthologues not only in mammals but also in lower vertebrates. A tBLASTn search, in which the amino acid sequence of human ART4 was compared to entries in NCBI's nucleotide sequence databases dynamically translated in all reading frames, resulted in the detection of a predicted gene transcript in chicken that potentially encoded a protein "similar to Dombrock blood group carrier molecule". Intriguingly, this protein contained an intact R-S-EXE motif, which raised the possibility that it represented an active ART enzyme. As none of the ARTs identified in chicken so far [13,14] was evolutionary related to mammalian ARTs, it was important to verify the orthologous relationship between the putative chicken and the human ART4 gene, before attempting to prove the enzymatic activity of its protein.

Here we report for the first time the existence of a GPI-anchored ART4 in chicken, which, to our knowledge, represents also the first real orthologue of a member of the mammalian ecto-ART family in a non-mammalian species. Moreover, in contrast to its mammalian ART4 orthologues, chicken ART4 displays an arginine-specific mono-ADP-ribosyltransferase activity.

## Methods

### Materials

[^32^P]-NAD^+ ^(800 Ci/mmole) was obtained from PerkinElmer LAS GmbH (Rodgau-Jugesheim, Germany). Oligonucleotides were synthesized by Invitrogen GmbH (Karlsruhe, Germany). Unless otherwise indicated materials used in this study were from the following manufacturers: Fermentas GmbH (St. Leon-Rot, Germany): *E. coli *DNA polymerase I, T4 DNA polymerase, T4 DNA ligase, *Pfu*-DNA polymerase, dNTP solution, RevertAid™ H Minus M-MuLV reverse transcriptase, High Fidelity PCR Enzyme Mix, and CloneJET™PCR Cloning Kit; Qiagen (Hilden, Germany): RNeasy Mini Kit; Invitrogen GmbH (Karlsruhe, Germany): TOPO TA Cloning Kit, 100 bp DNA ladder, pSecTagB plasmid, and Zeocin™; NEB GmbH (Frankfurt/Main, Germany): RNase H, Quick Ligation Kit, PNGase F; Sigma-Aldrich GmbH (Taufkirchen, Germany): 1,*N*^6^-etheno-NAD^+^, Anti-Flag^® ^M2 antibody, Anti-Flag^® ^M2 Affinity Gel, poly-L-arginine (molecular weight 5000 – 15000).

Sequencing was performed by GATC Biotech AG (Konstanz, Germany).

The animals of the study were neither infected nor manipulated otherwise and were kept as well as sacrificed considering the animal welfare and the International Guiding Principles for Biomedical Research. The chicks served exclusively as organ donors. Therefore, no permission was required for the use of the animals as regularised in §4 (3) of the German Animal Welfare Act.

### Cell culture and transfection

C-33A cells (human cervix carcinoma), a kind gift from Dr. Kurt Engeland (Frauenklinik, University of Leipzig, Germany) were cultured in Dulbecco's modified Eagle's medium (DMEM) supplemented with 10% (v/v) fetal bovine serum, 2 mM L-glutamine and antibiotics at 37°C and 10% CO_2_. Transient transfections of C-33A cells were performed using 4 μl FuGENE^® ^HD (Roche Diagnostics GmbH, Mannheim, Germany) according to the manufacturer's instruction. Exponentially growing cells (3.5 × 10^5^/well) were plated in 2 ml culture medium in 6 well plates. After 24 h they were transfected with 2 μg expression plasmids or 2 μg empty plasmids as a control. To obtain stably transfected cells they were incubated for four weeks with 250 μg/ml Zeocin™. After staining with the anti-human-ART4 or anti-Flag M2 antibodies high positive cells were enriched using a cell sorter (Becton Dickinson).

HD3 cells (chicken erythroblasts), a kind gift from Dr. Thomas Göbel (Institute for Animal Physiology, University of Munich) were cultured in RPMI 1640 medium supplemented with 8% (v/v) fetal bovine serum, 2% (v/v) chicken serum, 2 mM L-glutamine and antibiotics at 37°C and 5% CO_2_. Cells were maintained as suspension cultures (1 × 10^5 ^cells/ml) and after three to four days cells were passaged by 1:10 dilution with fresh culture medium.

Human embryonic kidney (HEK-293-T) cells, a kind gift from Dr. Friedemann Horn (Molecular Immunology, University of Leipzig, Germany) were grown in Dulbecco's modified Eagle's medium (DMEM) supplemented with 10% (v/v) fetal bovine serum, 2 mM L-glutamine and antibiotics at 37°C and 10% CO_2_. Cells were transfected as described [10]. To obtain stably transfected cells they were incubated for four weeks with 250 μg/ml Zeocin™.

### RNA isolation and reverse transcription

Total RNA was isolated from pieces of yolk sack (14 day old embryo, breed: Italian), from chicken bone marrow cells (2 × 10^6^) (breed: German White Leghorn; German White Leghorn × Rhode Island Red) or from HD3 cells (2 × 10^6^) using the RNeasy Mini Kit according to the manufacturer's instruction. Reverse transcription was performed as described previously [9].

### PCR primers and PCR reaction

Unless otherwise described, Table 1 shows the sequences of primers used in this study. PCR analysis (30 – 40 cycles) were carried out using cDNA from HD3 cells (chART4 fwd II/chART4 rev II or chART4 RACE3/3'UTR rev5) to amplify the chicken *ART4 *mRNA. PCR products were separated by electrophoresis on 1.5% (w/v) agarose gels (FMC Bioproducts, Rockland, MA, U.S.A.) containing 1.25 μg/ml ethidium bromide and visualized under UV light.

**Table 1:** List of primers used in this study

Primer	Sequence (5' – 3')
oligo dT_20_	TTT TTT TTT TTT TTT TTT TT
chART4 fwd II	GTC CTT GGG AGG ACT CCA G
chART4 rev II	AGA AAG CCA AGC AGT TCG TC
chART4 RACE3	TGG GGA ACT ACA GCA AGT ACC
3'UTR rev5	GGC CTT TTG TTC CTG AAG AG
RT_2_chART4	TTT CCC TAC GTT GTC CTT GC
chART4 inverse fwd1	ACA TCC ACA GCA GGA AGG TC
RT_chART4	AGC TTC CTG AGC CTT CTT CC
chART4 inverse fwd2	ACC TAA CGA CAG CCA TCC AG
chART4 inverse rev2	AAA TAG TCT CCC CGC TCC AG
RT-RACE-chick	GAT CTA GAG GTA CCG GAT CCT TTT TTT TTT TTT TTT TTV N
RACE reverse II	GAT CTA GAG GTA CCG GAT CC
chART4 Poly for	TGA GAA TTT TTG CCC AGC TC
chART4 Poly rev	AGA AAG CCA AGC AGT TCG TC
3'UTR fwd2	CCT TCA GCA GCC TTT CTT TG
3'UTR fwd3	TGC AGA TGG GTT ATT TTC ACC
3'UTR fwd4	ACG GAA GTC TTC ACA TGT CC
GAPDH fwd	GTC AGC AAT GCA TCG TGC A
GAPDH rev	GGC ATG GAC AGT GGT CAT AAG A
chA4 realtime 3	GTC CTC ATT CCC CCT TAT GA
chA4 realtime 5	TTC TTG ATT CTT GAA GCT TC

### 5' inverse RACE-PCR

First strand synthesis was performed with total RNA isolated from the yolk sack of a 14 day old embryo. A total of 10 μl (approx. 4 μg) was used to prepare cDNA by annealing RNA with 1 μl (100 pmol) of gene-specific primer RT_2_chART4. After denaturation for 10 min at 70°C reverse transcription using RevertAid™ H Minus M-MuLV reverse transcriptase at 37°C for 60 min was performed. An inactivation phase of 10 min at 70°C was followed by second strand synthesis, ring ligation, and inverse nested PCR amplification (primer pairs chART4 inverse fwd1/RT_chART4 and chART4 inverse fwd2/chART4 inverse rev2 as described previously [15].

### 3' RACE-PCR

To amplify the 3'-end of chicken *ART4 *mRNA from HD3 cells, 3'RACE-PCR was performed according to Frohman et al. [16]. Briefly, 10 μl total RNA (approx. 4 μg) were reverse transcribed for 60 min at 37°C using RevertAid™ H Minus M-MuLV reverse transcriptase and the RT-RACE-chick primer. Nested PCR (40 cycles) was performed using an adaptor-specific primer (RACE reverse II) and different forward primers (3'UTR fwd2, 3'UTR fwd3, and 3'UTR fwd4). PCR products were diluted 1:10 prior to the next PCR step.

### Quantitative real-time RT-PCR

Different organs (lung, bone marrow, thymus, spleen, caecum, liver, bursa of Fabricius) from five-day-old chicken were stored in RNA-later (Qiagen, Hilden, Germany) until use. Total RNA was extracted using the RNeasy Mini Kit (Qiagen) and contaminating DNA was digested using the RNase-free DNase Set (Qiagen). RNA was eluted in 50 μl RNase-free water per 20 mg tissue, stored and analysed by spectral analysis (BioPhotometer, Eppendorf, Hamburg, Germany). Only samples with mRNA purity of about 2 (ratio E260/280) or above 2 (E260/230) were used. The quantity of mRNA was adjusted and the mRNA expression rates of *ART4 *(chA4 realtime3/chA4 realtime5) and *GAPDH *(GAPDH fwd/GAPDH rev) was determined for each bird using the QuantiTect™ SYBR^® ^Green one-step RT-PCR Kit (Qiagen). Amplification and detection of specific products were performed on a Mx3000P™ real-time PCR equipment (Stratagene, La Jolla, CA) using the following temperature-time profile: one cycle at 50°C for 30 min, 96°C for 15 min, and 45 cycles at 94°C for 30 s, 55°C for 30 s followed by 72°C for 30 s. To check the specificity of amplification products, the dissociation curve mode was used (one cycle at 95°C for 1 min, 55°C for 30 s and 95°C for 30 s) subsequent to amplification and the amplicons were sequenced. Final quantification was done using the comparative Ct-method and reported as relative gene expression to cDNA from bone marrow (calibrator). The threshold cycle number (Ct) was calculated and the levels of chicken *ART4 *expression were normalized to *GAPDH *using the formula 2^-ΔΔCt ^in which ΔΔCt = ΔCt (sample) - ΔCt (calibrator) with ΔCt as difference between Ct of target gene (chicken *ART4*) and Ct of housekeeping gene (*GAPDH*).

### Cloning and sequencing of PCR products

PCR products were excised from agarose gels. The fragments were cloned using the TOPO TA cloning kit or the CloneJET™PCR Cloning kit according to the manufacturer's instruction. Plasmid DNA was purified using a GFX™ Micro Plasmid Prep Kit (Amersham Biosciences, Munich, Germany) and sequenced on both strands.

### Construction of a chicken *ART4 *expression plasmid

DNA from 1 × 10^7 ^chicken bone marrow cells (German White Leghorn × Rhode Island Red) was isolated using standard procedures. The coding sequence of chicken *ART4 *was amplified using genomic DNA from chicken bone marrow cells with primers derived from [GenBank: AADN01052179]. PCRs were carried out using the High Fidelity PCR Enzyme Mix with primers amplifying the coding sequence of exon2 (fwd: 5'-*GAC GAT GAC AAG *TCC CAC CTT ATG ATG-3' and rev: 5'-CTT GAT TCT TGA AGC TTC CAA GAG CTG G-3') and exon3 (fwd: 5'-CCA GCT CTT GGA AGC TTC AAG AAT CAA G-3' and rev: GAC TCG AGT CAT TGC TTG GCC AAG CAC-3'). The resulting PCR products (the sequences that are underlined represent complementary regions) were fused in a second PCR reaction using the following primer pair (fwd: 5'-TTG GTA CC*G ACT ACA AGG ACG ACG ATG A*-3' and rev: 5'-GAC TCG AGT CAT TGC TTG GCC AAG CAC-3') and the High Fidelity PCR Enzyme Mix. The chicken ART4 N-terminal signal peptide was exchanged by the Flag-tag (DYKDDDDK) (sequences of the primers that encode the Flag-tag are highlighted in italics). The resulting PCR product was purified and used for *Acc*65I/*Xho*I cloning into the pSecTagB plasmid. The construct-encoded chicken ART4 protein sequence was identical to the predicted protein sequence of [GenBank: XM425453.1] except for a F267L polymorphism. The polymorphism ([GenBank: EU056570]) was located within the GPI-anchor signal peptide and should have no effect on the mature protein.

### Site-directed mutagenesis of human ART4

Following oligonucleotides were used to mutate the Y-S-KKE motif: Y187R_for 5'-GAG GTG CAT AGG AGG ACG AAG GAT-3' and Y187R_rev 5'-ATC CTT CGT CCT CCT ATG CAC CTC-3' for exchanging the tyrosine^187 ^by an arginine and K242E_for 5'-TTC TCC CTC GAG AAG GAA GTC TTG-3' and K242E_rev 5'-CAA GAC TTC CTT CTC GAG GGA GAA G-3' for exchanging the lysine^242 ^by a glutamate (the changes are underlined). PCR was performed using a human ART4 expression plasmid [10] as template (20 cycles at 57°C) and the following primer combinations: 1) Y187R_for and Expr_rev [10]; 2) K242E_for and Expr_rev; 3) Expr_3 [10] and Y187R_rev; 4) Expr_3 and K242E_rev. The products were separated on agarose gels, excised, and extracted from the gels. A second PCR was performed (20 cycles, 54°C) using the PCR products from combination 1 and 3 or combination 2 and 4 as templates together with Expr_3 and Expr_rev as primer. The resulting PCR products were cloned into the pcDNA3.1/Zeo (+) plasmid as described [10]. For each mutagenesis the insert was sequenced to verify the mutation and to exclude the presence of other mutations.

### Treatment of C-33A cells with bacterial phospholipase C

C-33A cells (4 × 10^7^/ml) transfected with the chicken *ART4 *containing plasmid or the empty plasmid (pSecTagB) were suspended in PBS containing 5 U/ml *Bacillus cereus *phosphatidylinositol-specific phospholipase C (PI-PLC) (Invitrogen GmbH, Karlsruhe, Germany) and incubated for 60 min at 37°C. Cells were washed three times prior to the detection of chicken ART by flow cytometry as described below.

### Treatment of chicken ART4 with PNGase F

The supernatant of PI-PLC-treated C-33A cells transfected with a chicken *ART4 *containing plasmid was incubated in the presence or absence of PNGase F (20000 U/ml) according to the manufacturer's instruction.

### Expression of soluble, recombinant chicken ART4

An N-terminal Flag-tagged chicken ART4 (amino acids 20 – 270) was cloned (*Asp718I*/*XhoI*) into the pSecTagB plasmid. HEK-293T cells were stably transfected with the plasmid and grown in serum-free Panserin PX40 medium (PAN-Biotech GmbH, Aidenbach, Germany) at 37°C and 10% CO_2_. After 3 – 4 days the supernatant was exchanged by fresh medium and purified using the Anti-Flag^® ^M2 affinity gel according to the manufacturer instructions. Fractions (1 ml) were lyophilized, reconstituted in 100 μl aqua dest., combined and dialyzed against PBS.

This crude purified recombinant chicken ART4 was used for the ADP-ribosylation filter assay.

### Determination of K_m _values

After incubating recombinant chART4 in the presence or absence of PNGase F (20000 U/ml) for 1 h, ADP-ribosyltransferase activity was measured. The reaction mixture contained PBS (pH 7.4), poly-L-arginine (1 mg/ml), chART4 (7 μg/ml) and etheno-NAD^+ ^(0.5, 1, 2, and 5 μM). Controls were run in the absence of chART4. The reaction was started by the addition of etheno-NAD^+^, and cleavage of etheno-NAD^+ ^was measured after 0.5, 1.5, 2.5, 3.5, 4.5, 5.5, 6.5, 8, 10, 12, 15, 20, and 30 min in the Fluorolog 3 spectrophotometer (Horiba Jobin Yvon GmbH, Munich, Germany) The excitation wavelength was 310 nm and the emission was recorded at 403 nm. The fluorescence intensity was expressed as photons per second (CPS).

### ADP-ribosylation filter assay

The assay was performed in a total of 100 μl reaction mixture containing PBS (pH 7.4), 1 mM ADP-ribose, 50 μM [^32^P]-NAD^+ ^(10 μCi/assay) in the presence or absence of 100 μg poly-L-arginine and recombinant chicken ART4 (0.7 μg) for 30 min at 37°C. The reactions were terminated by adding 50 μl of ice-cold BSA (5 mg/ml) followed by 1.2 ml 25% (w/v) trichloroacetic acid. After 45 min at 4°C the resulting precipitates were recovered by centrifugation (10 min at 3000 × g). The pellets were resuspended in 250 μl of 2 M KOH, precipitated again and collected on Whatman GF/C glass-fibre filters. After washing, filters were counted by liquid scintillation spectrometry.

### Treatment of cells with etheno-NAD^+^

Cells (1 × 10^7^/ml) were incubated in PBS for 30 min at 37°C with 200 μM of etheno-NAD^+^. After adding a 20-fold volume of PBS cells were washed three times before performing FACS- or Western Blot analysis.

### Antibodies and FACS analysis

The anti-Flag M2 antibody, IgG1 and IgG2a isotype control antibodies were from Sigma-Aldrich. The anti etheno-adenosine specific antibody 1G4 (IgG2a) [17] was kindly provided by Dr. Friedrich Koch-Nolte (University Hospital Eppendorf, Hamburg, Germany). Cells were incubated with the respective mAbs for 30 min at 4°C. After washing in PBS containing 10% Haemaccel^® ^(Hoechst, Frankfurt, Germany), and 0.1% sodium azide they were incubated for 30 min at 4°C with FITC-labelled goat-anti-mouse antibody (SIFIN, Berlin, Germany). After washing and fixation in 1% formaldehyde, cells were analysed on a FACScan flow cytometer (Becton Dickinson, San Jose, CA, U.S.A.).

### Western Blot analysis

Western Blot analysis was carried out as described previously [18]. Cells (1.5 × 10^7^/ml) were suspended in lysis buffer (50 mM Tris/HCl pH 7.5, 150 mM NaCl, 1% (v/v) NP-40, 0.5% (w/v) deoxycholate, 0.1% SDS (w/v) and cOmplete protease inhibitor cocktail (Roche, Mannheim, Germany)) and sonicated. Samples were run on a 12% SDS-polyacrylamid gel (MiniProtean II, BioRad GmbH) and transferred to polyvinylidene difluoride membranes (Amersham Biosciences, Munich, Germany). Membranes were probed with an anti-Flag M2 antibody (10 μg/ml), 1G4 antibody (1/500) or anti-β-actin antibody (1/5000; clone AC-74, Sigma-Aldrich) and detected with a POD-conjugated goat anti-mouse (1/20000 Sigma-Aldrich) secondary antibody using the Western Blotting Luminol Reagent (Santa Cruz Biotechnology, Santa Cruz, CA, U.S.A.) detection system.

### Data analysis

Following programs were used: the standard nucleotide-nucleotide BLAST [blastn] program [19] to screen the database of expressed sequence tags (dbEST) and the tBLASTn program to search translated nucleotide database using a protein query [20], SIM4 software [21] to align cDNA sequences with the genomic DNA sequence, CLUSTAL W software [22] for multiple amino acid alignment, and both SignalP 3.0 [23] and GPI-SOM [24] program for prediction of cellular localization and membrane anchorage, respectively.

## Results

### Identification of an *ART4*-related gene in chicken

**Figure 1:**
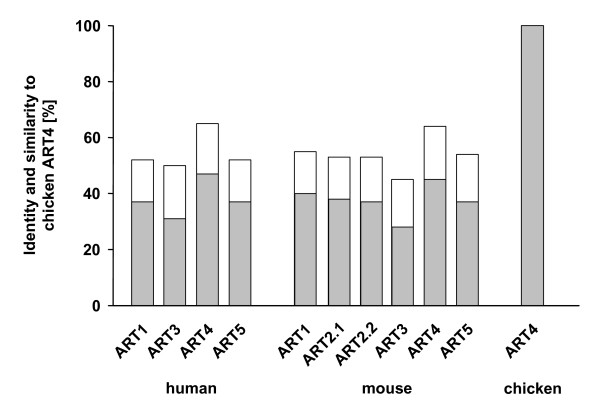
**Percentage amino acid similarity between members of the human and mouse ART family and chicken ART4**. The BLASTP program [25] was used to compare the amino acid sequences of the chicken protein with that of each member of the ART family in men and mice. Shown are the % identity (grey bars) and similarity (identity and conservative substitutions; white bars) of the proteins.

**Figure 2:**
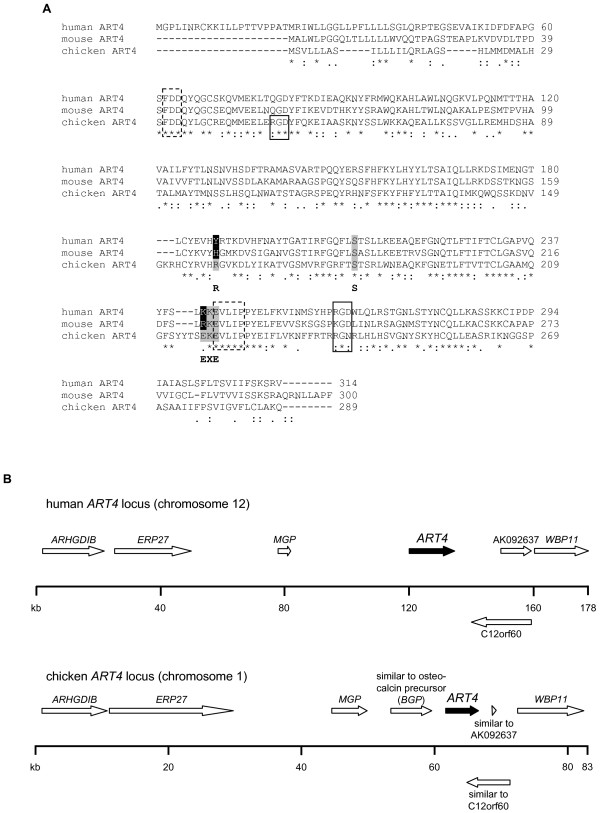
**Amino acid sequence alignment of ART4 from men, mice and chicken**. A) The deposited amino acid sequences from human ([GenBank: NM021071]), mouse ([GenBank: NM026639.2]) and chicken ART4 ([GenBank: XM425453.1]) were aligned using the CLUSTAL W (1.83) multiple sequence alignment program [22]. Residues corresponding to the R-S-EXE motif are shown in grey. Residues that differ from this motif are highlighted in white on black background. The numbering of the amino acids indicates the position of the single amino acids within the respective ART. Boxes indicate putative RGD sequence motifs (solid lines) or stretches of residues that are highly conserved among ARTs (broken lines). " * " marks residues that are identical in all sequences; " : " marks conserved substitutions and " . " marks semi-conserved substitutions. B) Comparison of the human and chicken *ART4 *loci. The genes are depicted as arrows with the *ART4 *genes shown as filled arrows. The gene order at the human and chicken loci was obtained from the NCBI map viewer

In order to locate *ART4*-related genes in species other than mammals, we made use of the tBLASTn program, which compares a protein query sequence to nucleotide sequences dynamically translated in all six reading frames. A search of the National Center for Biotechnology Information (NCBI) "nr" nucleotide database with the human or mouse ART4 protein sequence uncovered a putative chicken gene transcript of 870 nucleotides that translated into a 289 amino acid protein "similar to Dombrock blood group carrier molecule" ([GenBank: XM425453.1 GI:50729203, VRT 28-JUL-2004]). This record had been generated by "automated computational analysis" of a genomic contig ([GenBank: NW001471513]) on chicken chromosome 1, which resulted in the prediction of an *ART4*-related gene composed of three coding exons. As in mammalian *ART4 *genes, the second exon encoded the major part of the protein, but different from the situation in mammals, it also coded for an intact R-S-EXE motif indicative of a functional catalytic domain of an arginine-specific ADP-ribosyltransferase [6]. Incidentally, [GenBank: XM425453.1] was since removed from the "nr" database and on 06-Nov-2006 replaced by [GenBank: XM425453.2 GI:118082577]. (The protein encoded by the latter transcript differs at its C-terminus from the earlier version, because the new gene prediction lacks the third exon. As a consequence, the open reading frame in ex2 ends shortly after the ex2-intron2 junction predicted in the first record. Thus, [GenBank: XM425453.2] could reflect a splice variant.) To substantiate the presence of an active *ART4*-related gene in chicken, RT-PCR was performed on total RNA from HD3 cells (a chicken erythroblast cell line) with primers, designed to amplify the complete coding and additional flanking sequences of the trancript shown in [GenBank: XM425453.1]. A weak PCR-product of approx. 950 base pairs was obtained (data not shown, [GenBank: EU269858]). It corresponded to the transcript predicted in the original [GenBank: XM425453.1] entry, thus, verifying the existence of an avian *ART *gene with three coding exons as found in mammalian *ART4 *genes. Using the BLASTP program [25] to compare the amino acid sequence of the chicken protein with that of each member of the ART family in men and mice, the highest degree of sequence identity exhibited the ART4 orthologues, with 47% identity and 65% similarity between chicken and human or 45% identity and 64% similarity between chicken and mouse (Fig. 1). Figure 2A shows the actual amino acid sequence alignment of the putative chicken ART4 with its presumed counterparts in mouse and man. As noted before, only the chicken protein contains an intact R-S-EXE motif. Note that in all three ARTs the X within the EXE tripeptide is a lysine (K).

### Phylogentic relatedness of ART4 in chicken and mammals

Having demonstrated the existence of an apparently *ART4*-related gene in the chicken genome, we wanted to establish its phylogenetic relatedness with mammalian genes by analysing its location on chromosome 1 with respect to the presence and order of genes surrounding it. Employing NCBI's map viewer  and tBLASTn searches revealed that the chicken gene mapped to a region syntenic to the portion on human chromosome 12, which harbours the human *ART4 *gene (Fig. 2B). The analysed 83-kb *Gallus gallus *locus comprises seven genes in addition to *ART4*. Six of these genes were also found surrounding the human *ART4 *locus, whereas the gene (*BGP*) directly upstream of chicken *ART4 *had no human counterpart (Fig. 2B). The data presented so far strongly suggest that the predicted chicken gene is orthologous to mammalian *ART4 *genes.

### Analysis of the 5' and 3' untranslated region

To analyse the 5' end of the chicken *ART4 *mRNA we performed 5' inverse RACE-PCR on RNA derived from yolk sack. Among the respective PCR products we obtained a product of 422 bp (data not shown). According to the sequence analysis of the product ([GenBank: EF626644]) which includes exon 1 and parts of exon 2 the 5' UTR consists of 206 bp.

The 3' UTR of the chicken *ART4 *mRNA was analysed by applying 3' inverse RACE-PCR [26] or 3' RACE-PCR [16] on mRNA from HD3 cells using primers derived from the predicted *ART4 *sequence. As no *ART4 *specific transcripts were obtained, possibly due to the length of the products we applied conventional RT-PCR analysis to amplify *ART4 *mRNA reaching into the 3' UTR as far as possible. We obtained a PCR product ([GenBank: EU048538]) of 2199 bp (data not shown) consisting mainly of exon 3 and to a minor extent of exon 2. It contains three obviously unused polyadenylation signals. In a subsequent 3' RACE-PCR analysis using primers derived from the above described PCR product we detected a transcript (GenBank: EU048537]), representing the 3' end of the chicken *ART4 *mRNA.

**Figure 3:**
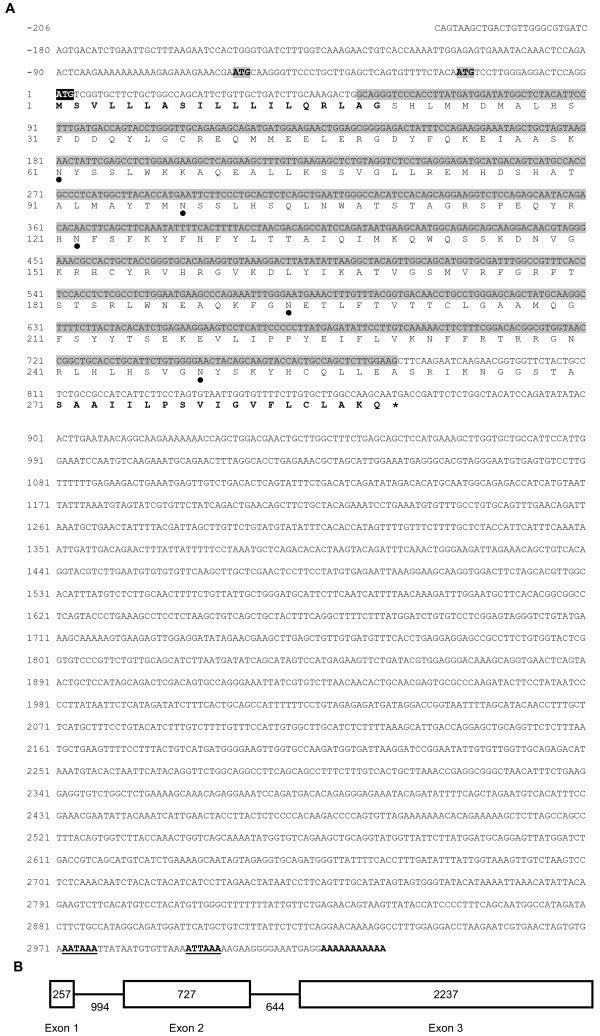
**Nucleotide- and deduced amino acid sequence of chicken *ART4***. A) The nucleotide sequence is numbered relative to the initiation codon that is represented by bold white letters on black background. The derived amino acid sequence is shown below the nucleotide sequence. Predicted sites for N-glycosylation are indicated by closed circles. The N- and C-terminal signal peptides are represented by bold letters. Asterisk indicates the stop codon. Exon 2 is highlighted in grey. ATG codons out of reading frame are indicated by bold black letters on grey background. AAA... (bold) represents the poly A tail of the mRNA and the polyadenylation signal is shown in bold and underlined. B) Schematic representation of the chicken *ART4 *gene. Exons are indicated by boxes, introns by lines. The lengths of exons and introns are indicated by numbers.

Figure 3A combines the genomic DNA and cDNA sequences ([GenBank: EF626644, EU048538, EU048537, and XM425453.1]) to depict the chicken *ART4 *mRNA. It shows that chicken ART4, as predicted by computer prediction programs [23,24], contains N- and C-terminal signal peptides, characteristic of extracellular GPI-anchored membrane proteins. The derived chicken *ART4 *gene structure is shown in Figure 3B.

### Chicken ART4 is a GPI-anchored and glycosylated protein

**Figure 4:**
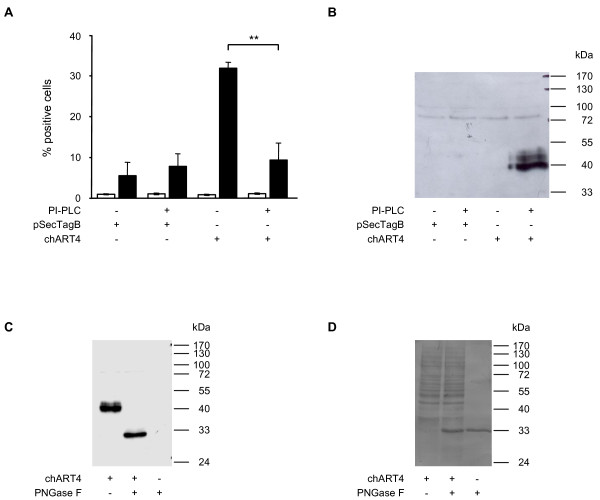
**Treatment of chicken *ART4 *transfected C-33A cells with PI-PLC and PNGase F**. A) C-33A cells (4 × 10^7^/ml) transfected with the chicken *ART4 *containing plasmid or the empty plasmid (pSecTag B) were stained with the anti-Flag antibody (black bar) or the isotype control (white bar) after being incubated for 1 h at 37°C in the presence or absence of PI-PLC (5 U/ml). Data show the mean of % positive cells ± S.E.M. of three experiments. ** p = 0.007 (Student's t-test) B) Western blot analysis using the anti-Flag antibody was performed with supernatants of chicken *ART4 *transfected C-33A cells after incubation in the presence or absence of PI-PLC. Data show one representative experiment out of three. C) The supernatant of chicken *ART4 *transfected cells was incubated for 1 h in the presence or absence of PNGase F (20000 U/ml) at 37°C. PNGase F alone was run as a control. After boiling the samples in SDS-PAGE sample buffer, Western blot analysis using the anti-Flag antibody were carried out. The membrane was stained with Ponceau S (D). Similar results were obtained in three separate experiments.

C-33A cells were stably transfected with an expression plasmid containing the sequence of *ART4 *encoding amino acids 20–289. As shown by FACS analysis about 30% of the stably transfected cells were fluorescence positive (Fig. 4A). Treatment with PI-PLC resulted in a significant decrease from 30% to 9.4% positive cells, indicating the existence of a GPI-anchored protein. Proteins of the supernatant of the PI-PLC treated C-33A cells were separated by SDS-PAGE and ART4 was detected by Western Blot analysis. The apparent molecular weight was 40 kDa, about 10 kDa higher than predicted (Fig. 4B). As chicken ART4 contains five potential N-glycosylation sites we tested whether the higher mass could be due to glycosylation of the protein. We incubated the chicken ART4 containing supernatant with PNGase F, an enzyme that cleaves the amide bonds between GlcNAc and asparagine residues of N-linked glycoproteins [27]. The molecular weight of ART4 detected in the supernatant by Western Blot analysis was about 10 kDa lower than the untreated protein (Fig. 4C), indicating the presence of asparagine-linked glycosyl moieties on chicken ART4. After having probed the membrane with the anti-Flag M2 antibody, the membrane was stained with Ponceau S. As seen in figure 4D there was hardly any difference between the molecular weight of chicken ART4 and PNGase F. However, the anti-Flag antibody only detected chicken ART4 so that any unspecific binding of the antibody to PNGase F can be excluded.

### Chicken ART4 is enzymatically active

**Figure 5:**
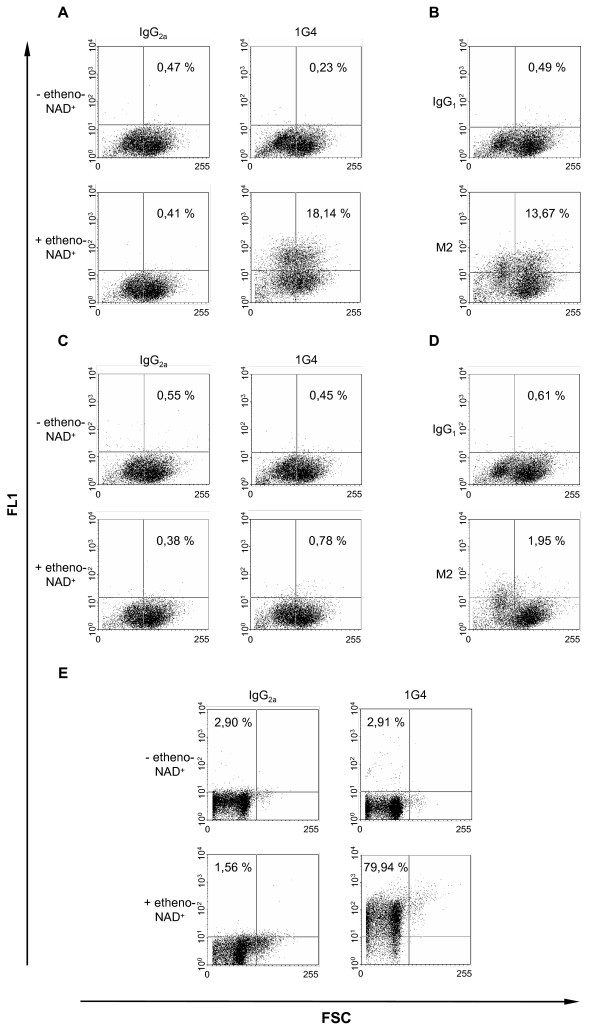
**Detection of ADP-ribosylated proteins by flow cytometry**. C-33A cells (1 × 10^7^/ml) transiently transfected with a Flag-tagged chicken *ART4 *containing plasmid (A, B) or the empty plasmid (pSecTag B) (C, D) were incubated at 37°C in the presence or absence of 200 μM etheno-NAD^+ ^for 30 min. After washing, cells were stained with an etheno-adenosine specific antibody (1G4) (A, C) or an anti-Flag antibody (M2) (B, D) and the respective isotype control. Numbers in the upper right quadrant indicate the percentage of fluorescence positive cells. Shown are typical scattergrams (fluorescence (FL1) vs. forward scatter (FSC)) of one representative experiment out of three. (E) Chicken erythrocytes (1% v/v), isolated by density gradient centrifugation, were treated as described above and stained with the 1G4 antibody. Shown is one representative experiment out of 8.

In contrast to human ART4 the chicken ART4 contains the R-S-EXE motif, a characteristic for arginine-specific ARTs [6]. ART4 activity was measured by incubating ART4 transfected C-33A cells with etheno-NAD^+^, an analogue of NAD^+^. Etheno-NAD^+ ^can be used as an alternative substrate to monitor etheno-ADP-ribosylation of cell surface proteins. The etheno-ADP-ribosylated proteins were visualized by flow cytometry using an etheno-adenosine specific antibody (1G4) [28]. As shown in Figure 5, transient transfection of C-33A cells with chicken ART4 leads to an ADP-ribosylation of cell surface proteins (Fig. 5A, C). Transfection efficiency was monitored by staining the cells with an anti-Flag antibody (Fig. 5B, D). About 14% of the cells were ART4 positive and about 18% carried ADP-ribosylated proteins. As seen in Figure 5E ADP-ribosylated proteins are also detectable on chicken erythrocytes indicating that ADP-ribosylation is not only mediated by recombinant ART4 but also occurs endogenously.

**Figure 6:**
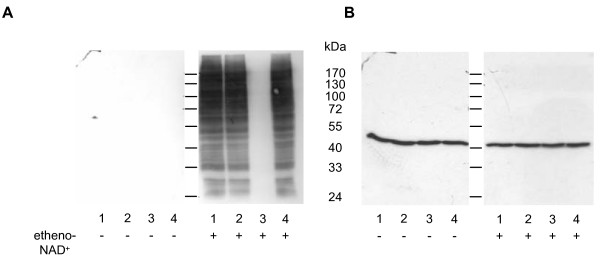
**Effect of NH_2_OH and HgCl2 on the stability of the ADP-ribose bond**. C-33A cells (1 × 10^7^/ml) transfected with the chicken *ART4 *containing plasmid were incubated at 37°C in the presence or absence of 200 μM etheno-NAD^+ ^for 30 min. After washing, cells were lysed and either directly precipitated (lane 1) or incubated at 37°C in the presence of 1 M NaCl (lane 2), 1 M NH_2_OH (pH 7.0) (lane 3) or 10 mM HgCl_2 _(lane 4). After 2 h proteins were precipitated with methanol. All samples were subjected to Western blot analysis using the etheno-adenosine specific antibody (A). After stripping, the blot was reprobed with a beta-actin specific antibody (B). Similar results were obtained in three separate experiments.

To characterize the nature of the linkage between ADP-ribose and amino acid side chains cells were incubated in the presence of etheno-NAD^+^. Cell lysates were prepared and incubated under conditions known to cleave specifically the linkage of ADP-ribose to thiol groups (10 mM HgCl_2_), or to arginine (1 M NH_2_OH, pH 7.0) [29,30]. SDS-PAGE followed by Western blot analysis using the 1G4 antibody [28] revealed that detection by the antibody was not influenced by treatment with 1 M NaCl (Fig. 6A, lane 2) and 10 mM HgCl_2 _(lane 4) but was abolished in the presence of 1 M NH_2_OH (lane 3) strongly suggesting that chicken ART4-catalyzed mono-ADP-ribosylation occurred at arginine residues. As a loading control, the membranes were stripped and reprobed with a β-actin specific antibody (Fig. 6B).

To verify that arginine-residues were modified by chicken ART4, ADP-ribosylation of poly-L-arginine was determined by incubating chicken ART4 in the presence of [^32^P]-NAD^+^, ADP-ribose and poly-L-arginine. ADP-ribose was added to minimize the contribution of the non-enzymatic addition of free [^32^P]-ADP-ribose to poly-L-arginine.

**Figure 7:**
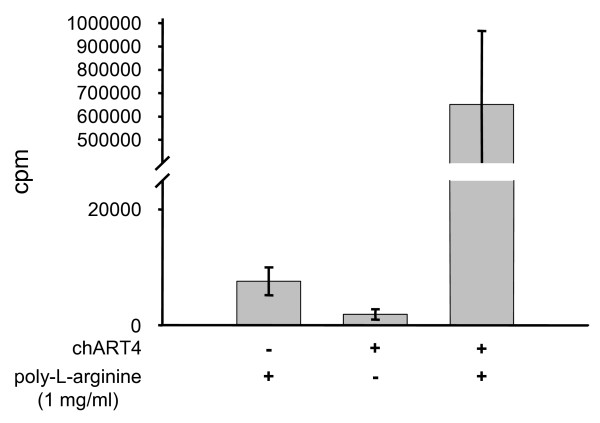
**ADP-ribosylation of poly-L-arginine**. Recombinant chicken ART4 (0.7 μg) was incubated in 100 μl PBS containing 1 mM ADP-ribose, 50 μM [^32^P]-NAD^+ ^(10 μCi/assay) in the presence or absence of poly-L-arginine (1 mg/ml) for 30 min at 37°C. After incubation the samples were precipitated and collected on Whatman glass-fibre filters as described in the materials and methods. Incorporated radioactivity was measured by liquid scintillation counting. Data show the mean ± S.D. (n = 3).

The finding that radioactivity was incorporated in poly-L-arginine (Fig. 7) demonstrates that ART4 catalyzed mono-ADP-ribosylation occurred at arginine residues.

**Figure 8:**
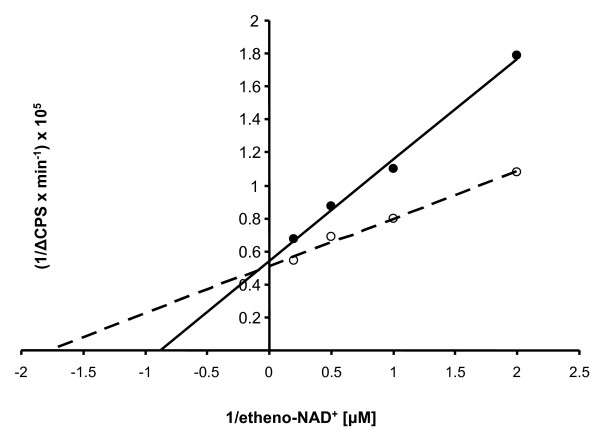
**K_m _determination for etheno-NAD^+ ^of chicken ART4**. Recombinant chART4 (7 μg/ml) was incubated at room temperature for 0.5, 1.5, 2.5, 3.5, 4.5, 5.5, 6.5, 8, 10, 12, 15, 20, and 30 min in the presence of poly-L-arginine (1 mg/ml) and 0.5, 1, 2, and 5 μM etheno-NAD^+ ^in the presence (open circles) and absence (filled circles) of PNGase F. The ADP-ribosyltransferase activity was determined by measuring the increase in fluorescence intensity. Shown is a double reciprocal plot of the data of the dose response. Data show one representative experiment out of three (K_m _"untreated" vs. K_m _"treated" chART4 p = 0.062, Student's t-test).

To further characterize chicken ART4 the K_m _value of the enzyme was determined. Recombinant chART4 was used for time- and dose-dependent determinations of enzymatic activity. Lineweaver-Burk analysis revealed an apparent K_m _for etheno-NAD^+ ^of 1.0 ± 0.18 μM (mean ± S.D., n = 3) of recombinant chART4 (Fig. 8). Treatment of the protein with PNGase F resulted in a decrease in the K_m _value (0.7 ± 0.12 μM; mean ± S.D., n = 3) which was not significantly different from the K_m _value of the untreated protein (p = 0.062, Student's t-test).

### Mutation of the active site motif of human ART4

To test whether human ART4 containing an R-S-EXE motif is enzymatically active three mutant proteins were constructed and tested for ADP-ribosyltransferase activity using etheno-NAD^+ ^as substrate. Expression plasmids were generated which encoded for the wildtype ART4, an Y187R mutant, a K242E mutant, and an Y187R, K242E double mutant containing the R-S-EXE motif. C-33A cells were stably transfected with the plasmids and those cells carrying either the wildtype ART4, the single mutants or the double mutant were enriched by cell sorting (more than 90% positive cells) and used for further analyses. FACS-analysis revealed that neither the mutants carrying a single amino acid exchange nor the double mutant (R-S-EXE) displayed any enzyme activity (data not shown). Obviously additional residues besides the intact R-S-EXE motif are involved in catalyzing the enzymatic reaction.

### Tissue distribution of chicken *ART4*

**Figure 9:**
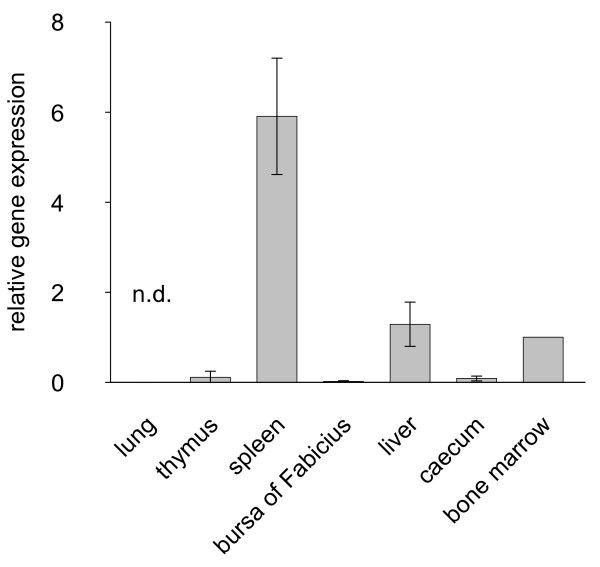
**Real-time RT-PCR analysis of chicken *ART4 *mRNA expression in different tissues**. The mRNA was isolated and mRNA levels of chicken *ART4 *were measured by quantitative real-time RT-PCR. Levels were standardized to the expression of GAPDH and mRNA concentrations from bone marrow were set as the 100% reference. Data show the mean ± S.D. of relative mRNA levels of two individual animals. n.d.: not detectable

Quantitative real-time RT-PCR analysis of chicken tissues revealed that the highest amount of chicken *ART4 *mRNA was present in spleen, liver and bone marrow, while in thymus, bursa of Fabricius, and caecum it was hardly detected. No mRNA was detected in lung (Fig. 9).

## Discussion

Whereas most studies have focussed on ART genes and activity in mammals expression of ARTs has also been demonstrated in chicken. Chicken contain both soluble ARTs (ART6.1 and ART6.2) and GPI-anchored ARTs (cgART1 and cgART2). The nucleotide sequence of *cgART2 *was found to be identical with that of *ART7 *(*ART7.2*) and the cDNA of *cgART1 *encoded a polypeptide which represents an *ART7 *homologue (*ART7.1*) [31]. The GPI-anchored ARTs showed different enzymatic properties than soluble ARTs and the tissue distribution of cgART1 and cgART2 revealed distinct expression patterns [31]. Besides ART6 and ART7 no other ARTs have been identified in chicken so far. Here we show that apart from ART6 and ART7 chicken also express a non chicken-specific ART.

We describe the cloning and characterization of the *ART4 *(or *DO*) gene from *Gallus gallus*. Several lines of evidence, such as conserved synteny, similar exon-intron structure, and conserved residues show that the gene cloned is the *Gallus gallus *orthologue of the human *ART4 *gene. The predicted chicken ART4 amino acid sequence only shares 47% and 45% identity (65% and 64% similarity) to human and mouse ART4 respectively and among mammals ART4 also appears to be quite divergent with an identity between human and mouse ART4 of 63% (75% similarity). Thus the coding sequence of *ART4 *appears to be evolving rapidly. Residues that are conserved during evolution in such a rapidly evolving gene or gene family are likely to be important for the function of the protein.

The amino acid sequence alignment of ART4 from chicken, men, and mice uncovered several stretches of residues that are highly conserved, most strikingly the S^30^FDDQY^35 ^and K^218^EVLIPPYE^226 ^residues (Fig. 2A). Parts of these sequences namely the FDD and EVLIP motifs are also strictly conserved within other members of the ART family. The FDD sequence motif is located in the N-terminus of the ARTs whereas the EVLIP stretch resides in the 5^th ^of the six conserved β-strands that make up part of the core features of the ART fold in the catalytic domain. Moreover a lysine residue (K) directly upstream of the catalytic E in the EXE motif is found in ART4 of men (KKE^244^), mice (RKE^223^), and chicken (EKE^219^) but not in the EXE motif of other mammalian ARTs [6].

Another common feature of the chicken and human *ART4 *gene is their genomic organization. Almost the complete mature protein is encoded by exon 2, one of the three exons that besides two introns make up the *ART4 *gene [10]. Unlike human *ART4 *mRNA the 3' UTR of the chicken *ART4 *mRNA comprises 2145 bp. Due to this length the chicken *ART4 *gene overlaps with a hypothetical gene ([GenBank: XM416185]) located on the opposite DNA strand.

The tissue distribution of chicken *ART4 *mRNA expression is in part similar to that of human *ART4 *mRNA [6,7]. Haematological tissues including human fetal liver and human and chicken bone marrow and spleen seem to be preferential sites of *ART4 *mRNA expression. Furthermore, the fact that HEL cells, a human erythroleukaemia cell line [10] and human erythrocytes carry the ART4 protein and that *ART4 *mRNA is present in chicken erythrocytes (data not shown) may point to a role of ART4 in haematopoiesis in higher vertebrates.

GPI-anchoring of the chicken ART4 to the membrane was shown by PI-PLC sensitivity. Treatment of chicken *ART4 *transfected C-33A cells with the enzyme led to the appearance of a 40 kDa protein (Fig. 4B) whose molecular weight was higher than that predicted for chicken ART4 (29 kDa) lacking the N- and C-terminal signal peptides. The decrease in molecular weight after the exposure to PNGase F strongly suggests that the chicken ART4 is glycosylated. In fact there are five predicted N-glycosylation sites in the amino acid sequence (Fig. 3A).

Determination of the apparent K_m _for etheno-NAD^+ ^of recombinant chicken ART4 revealed a value of 1.0 μM. It is similar to the K_m _(3 μM) for etheno-NAD^+ ^of ART2.2 expressed on DC27.10 cells [28] but it differs from the K_m _values for recombinant ART7.2 (130 μM) [32] another ART found in chicken and *Pseudomonas aeruginosa *toxin A (275 μM) [33].

Interestingly, both human and chicken ART4 contain an RGD sequence motif. This sequence (Arg-Gly-Asp) found in various adhesive proteins (fibronectin, vitronectin, fibrinogen, and Willebrand factor) serves as a cell attachment site [34]. In chicken the motif (RGD^50^) is found in the N-terminal part of ART4 whereas in humans an RGD^265 ^motif is located in the C-terminus that aligns to the chicken RGN^240^. In case ART4 should display adhesive properties it would be of interest in how far the cell recognition signal Arg-Gly-Asp is involved in it.

We show that chicken ART4 contains residues corresponding to the R-S-EXE active site motif of arginine-specific ARTs [6] and that it displays arginine-specific enzyme activity while human ART4 shows nonconservative amino acid deviations in this motif. Furthermore, according to the CLUSTAL W amino acid sequence alignment human and mouse ART4 not only deviate in the glutamic acid (E) two residues upstream of the catalytic glutamic acid (E) of the R-S-EXE motif but also in the arginine (R) which is replaced by histidine (H) in mouse and tyrosine (Y) in men (Fig. 2A).

Considering the importance of the R-S-EXE motif in the catalytic core of all arginine modifying ARTs we exchanged two amino acids of the human ART4 Y-S-KXE motif to obtain an intact R-S-EXE motif. Surprisingly C-33A cells expressing human ART4 with the R-S-EXE motif did not display any enzyme activity. Obviously the mere presence of the correct motif in ART4 is not sufficient to turn the protein into an active enzyme. Further mutational studies are necessary to identify additional amino acid residues essential for restoring a functional catalytic core.

It is conceivable that the common ancestor of birds and mammals possesses an enzymatically active ART4 and that the loss of enzyme activity of human ART4 may have arisen due to some evolutionary advantages.

Having cloned and characterized an enzymatically active *ART4 *orthologue we have provided important experimental tools to elucidate the functional role of ART4. Knowledge of such function in turn may help to explain the loss of ART4 enzyme activity in mammals during evolution.

## Conclusion

Mono-ADP-ribosyltransferases (ARTs) catalyze the transfer of a single ADP-ribose moiety of NAD^+ ^to a specific amino acid in a target protein. Mammalian ARTs consist of five members (ART1 – 5) two of them ART3 and ART4 being enzymatically inactive, unlike the active members they do not contain the R-S-EXE motif in the catalytic domain typical of arginine-specific ADP-ribosyltransferases. Here we identified the chicken ART4 as the first real orthologue of a member of the mammalian ARTs in a non-mammalian species. In contrast to the mammalian ART4 the chicken ART4 contains an intact R-S-EXE motif and exhibits the predicted enzyme activity. Thus, ART4 is the first ADP-ribosyltransferase which displays different biochemical properties in two higher vertebrate classes.

## Authors' contributions

AG performed the sequence alignments, carried out the protein and enzymatic experiments, participated in the mRNA analyses and helped to draft the manuscript. SR carried out the mRNA analyses and AB the real time RT-PCR analyses. FS performed the mutational analyses of human ART4. SH supervised and designed the study with essential contribution by AG, participated in its coordination, and drafted the manuscript. All authors read and approved the final manuscript.
